# Diurnal variations in CO_2_ exchange fluxes and their influencing factors in a shallow macrophyte-dominated lake in the northeastern Qinghai-Tibetan Plateau: a case study of Hurleg Lake

**DOI:** 10.3389/fpls.2025.1721578

**Published:** 2025-12-10

**Authors:** Junxiang Xie, Yanxiang Jin, Xin Jin, Zi’ang Li, Tongrui Zhang, Xin Zhang, Jingyun Yang

**Affiliations:** 1 Qinghai Province Key Laboratory of Physical Geography and Environmental Process, College of Geographical Science, Qinghai Normal University, Xining, China; 2 Key Laboratory of Tibetan Plateau Land Surface Processes and Ecological Conservation (Ministry of Education), Qinghai Normal University, Xining, China; 3 Academy of Plateau Science and Sustainability, People’s Government of Qinghai Province and Beijing Normal University, Xining, China

**Keywords:** Qinghai-Tibetan Plateau, shallow macrophyte-dominated lake, carbon cycle, water-air interface, water-sediment interface, carbon dioxide

## Abstract

As an important component of inland waters, shallow macrophyte-dominated lakes significantly influence regional carbon budgets. By using the static chamber-gas chromatography method and the sediment in-situ simulation, continuous fixed-point observations of CO2 exchange fluxes (F(CO2)) at water-air and water-sediment interfaces of shallow macrophyte-dominated Hurleg Lake were conducted. Combination with watershed meteorological conditions and lake water environmental parameters, their influencing factors were explored. The results revealed significant diel variations in F(CO_₂_) at both interfaces, characterized by peaks in the early morning and troughs in the evening or late night-a common feature of shallow macrophyte-dominated lakes. The composition of submerged macrophyte communities considerably affected the relative contribution of sediment-released CO₂ to the net atmospheric flux. The maximum contribution was observed in areas dominated by Potamogeton, followed by Myriophyllum zones, while the minimum occurred in Chara beds. Nocturnal F(CO_₂_) played a critical role in sustaining the carbon source function of the lake, accounting for 22.65%–42.90% of the total daily flux at the water-air interface and 5.57%-64.54% at the water-sediment interface across different vegetated and unvegetated zones. Neglecting nocturnal F(CO_₂_) would substantially increase uncertainties in estimating the lake’s overall carbon budget. The F(CO_₂_) at the water-air interface was primarily regulated by water temperature, pH, dissolved oxygen, and atmospheric pressure, whereas F(CO_₂_) at the sediment-water was mainly driven by porewater CO_₂_ concentration, sediment porosity, and water temperature.

## Introduction

**Figure 1:**
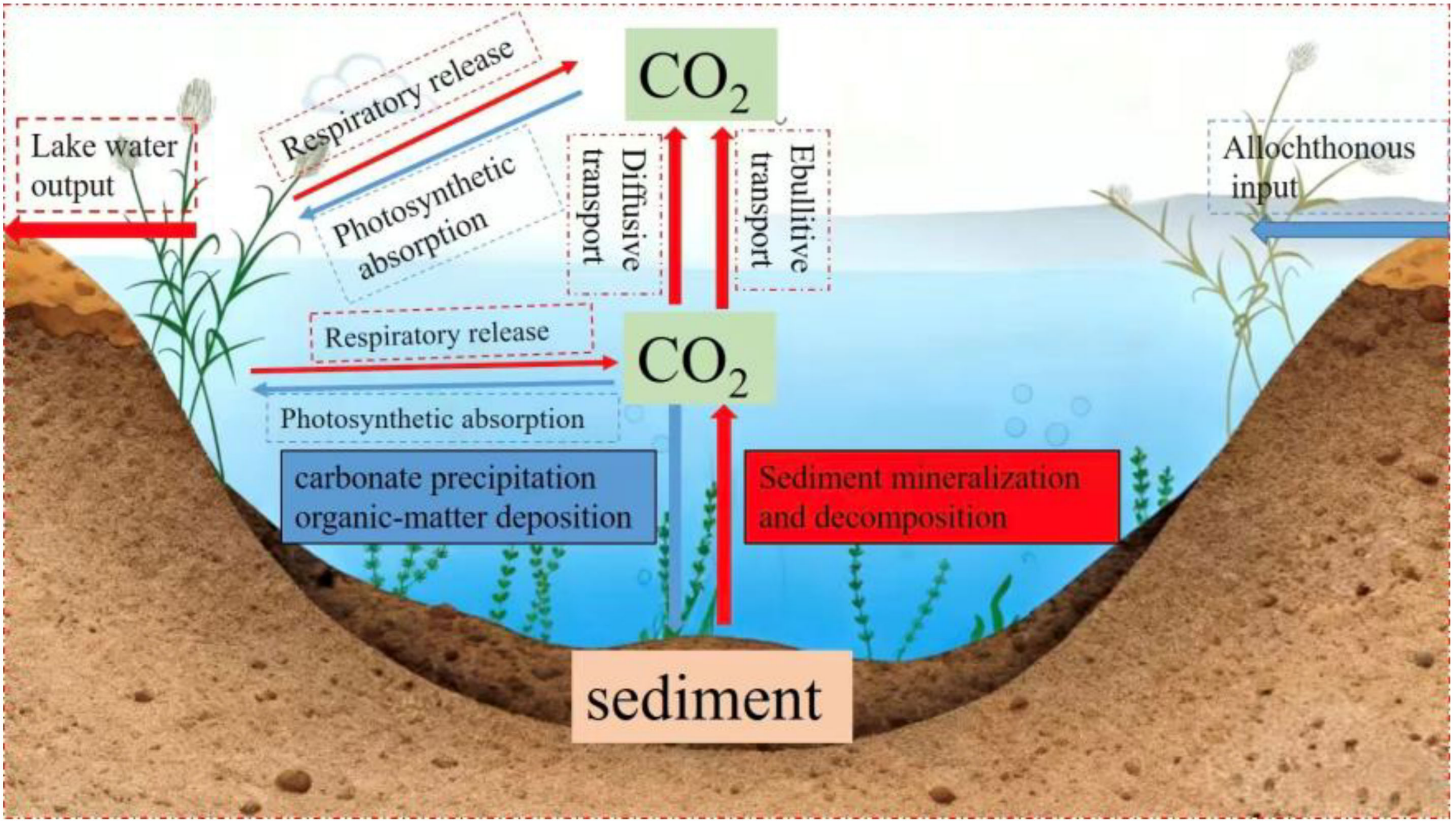
Schematic diagram of the lake carbon cycle (Revised from Duan et al., 2021) (Abbreviations: DIC, dissolved inorganic carbon; DOC, dissolved organic carbon; PIC, particulate inorganic carbon; POC, particulate organic carbon; CO2, carbon dioxide).

CO_2_ is the most significant greenhouse gas, contributing nearly 75% to the global greenhouse effect (Rocher-Ros et al., 2023; Chen et al., 2024). Lakes, covering only 3.7% of the global land area, are crucial sites for the release, storage, and transformation of carbon, making them important ecosystems influencing the global carbon budget (Verpoorter et al., 2014) (**Figure 1**). As a special type of lake, shallow macrophyte-dominated lakes typically exhibit highly primary productivity and abundant aquatic vegetation. The greenhouse gas exchange processes at water-air and water-sediment interfaces directly affect the lake’s carbon budget function (Sepulveda-Jauregui et al., 2015). A deep understanding of these exchange processes in shallow macrophyte-dominated lakes is essential for accurately assessing the role of lakes in the global carbon cycle.

The Qinghai-Tibetan Plateau has a unique climate and environment, hosting diverse lakes including freshwater, brackish, and saline lakes. Their number accounts for 41% of China’s total lakes, and their area covers about 57% (Zhang et al., 2019). Shallow macrophyte-dominated lakes are widely distributed in this region, and their carbon budget changes significantly impact regional and global carbon cycles (Yao et al., 2022). In recent years, the process of greenhouse gas exchange at the water-air interface of lakes on the Qinghai-Tibetan Plateau and its influencing factors have received widespread attention from researchers. However, research on this specific type, shallow macrophyte-dominated lakes, is relatively scarce, particularly regarding their diel dynamic characteristics and water-sediment interface processes.

Li et al. (2018) monitored the greenhouse gas exchange flux at the water-air interface of Qinghai Lake from 2013–2017 using the eddy covariance method, indicating that saline lakes on the Qinghai-Tibetan Plateau are significant carbon sinks, primarily attributed to chemical absorption processes associated with high pH values. Wang et al. (2022) conducted continuous monitoring of CO_2_ exchange fluxes at the water-air interface of Qinghai Lake and Hala Lake from 12:00-16:00 on October 20 and 23, 2018, using the headspace equilibrium method, finding both lakes to be sinks for atmospheric CO_2_; they identified salinity, dissolved organic matter (DOC), temperature, and dissolved oxygen (DO) as the main factors influencing CO_2_ exchange fluxes at the water-air interface of lakes on the Qinghai-Tibetan Plateau (Liu et al., 2024). Conversely, Ran et al. (2021) estimated the partial pressure of CO_2_ (ρCO_2_) in lakes on the Qinghai-Tibetan Plateau based on lake water chemistry data and found lakes to be carbon sources (Gang et al., 2023). Yan et al. (2018) instantaneously monitored greenhouse gas concentrations in 17 lakes on the Qinghai-Tibetan Plateau using the headspace equilibrium-gas chromatography method, discovering that the littoral area is a source of greenhouse gases, and its emission rate might be influenced by DOC concentration and water temperature. Jia et al. (2022) integrated instantaneously monitored lake CO_2_ exchange flux and ρCO_2_ data from the Qinghai-Tibetan Plateau from 2000-2020, indicating that lakes were carbon sources during this period, but would act as carbon sinks when the ice-covered period was included in carbon estimates. Clearly, whether lakes on the Qinghai-Tibetan Plateau are carbon sources or sinks remains uncertain across different spatiotemporal scales, and research on this specific type, shallow macrophyte-dominated lakes, is even more lacking. On one hand, the complex production/consumption mechanisms on the shallow macrophyte-dominated lakes lead to complexity in the greenhouse gas exchange process at the water-air interface (Lin, 2024). On the other hand, greenhouse gas exchange processes in different water types within shallow macrophyte-dominated lakes and in different areas of the same water body show significant spatiotemporal differences Gruca-Rokosz and Tomaszek, 2011; Ye et al., 2019). Furthermore, differences in monitoring methods, sampling time and spatial scales, as well as lake type and size, also increase the uncertainty in estimating the carbon budget of lakes in this region. Therefore, long-term, continuous monitoring of greenhouse gas exchange fluxes at the water-air interface of shallow macrophyte-dominated lakes is necessary to deeply understand the lake carbon budget and reduce the uncertainty in lake carbon budget estimation.

Compared to the water-air interface, research on the greenhouse gas exchange process at the water-sediment interface of shallow macrophyte-dominated lakes on the Qinghai-Tibetan Plateau is even more scarce. Existing studies on water-sediment interface greenhouse gases mainly focus on reservoirs, estuaries, and urban rivers (Sun, 2022). In fact, the diffusive flux of greenhouse gases at the lake water-sediment interface can significantly promote the release process of greenhouse gases at the water-air interface (Wen et al., 2017; Zhang and Gao, 2021). Therefore, we urgently need to conduct research on the greenhouse gas exchange process at the water-sediment interface of shallow macrophyte-dominated lakes on the Qinghai-Tibetan Plateau to gain a deeper understanding of the carbon budget in lakes.

This paper selects Hurleg Lake, a typical shallow macrophyte-dominated lake in the northeastern Qinghai-Tibetan Plateau, as the study area. Continuous diel monitoring of CO_2_ exchange fluxes at the water-air and water-sediment interfaces was conducted by using the static chamber-gas chromatography method and the sediment incubation method. The characteristics of greenhouse gas exchange at the water-air and water-sediment interfaces of shallow macrophyte-dominated lakes and their influencing factors are discussed. This provides an empirical case for research on the carbon budget of shallow macrophyte-dominated lakes on the Qinghai-Tibetan Plateau and helps to improve the accuracy of carbon budget estimation.

## Materials and methods

### Study area

**Figure 2:**
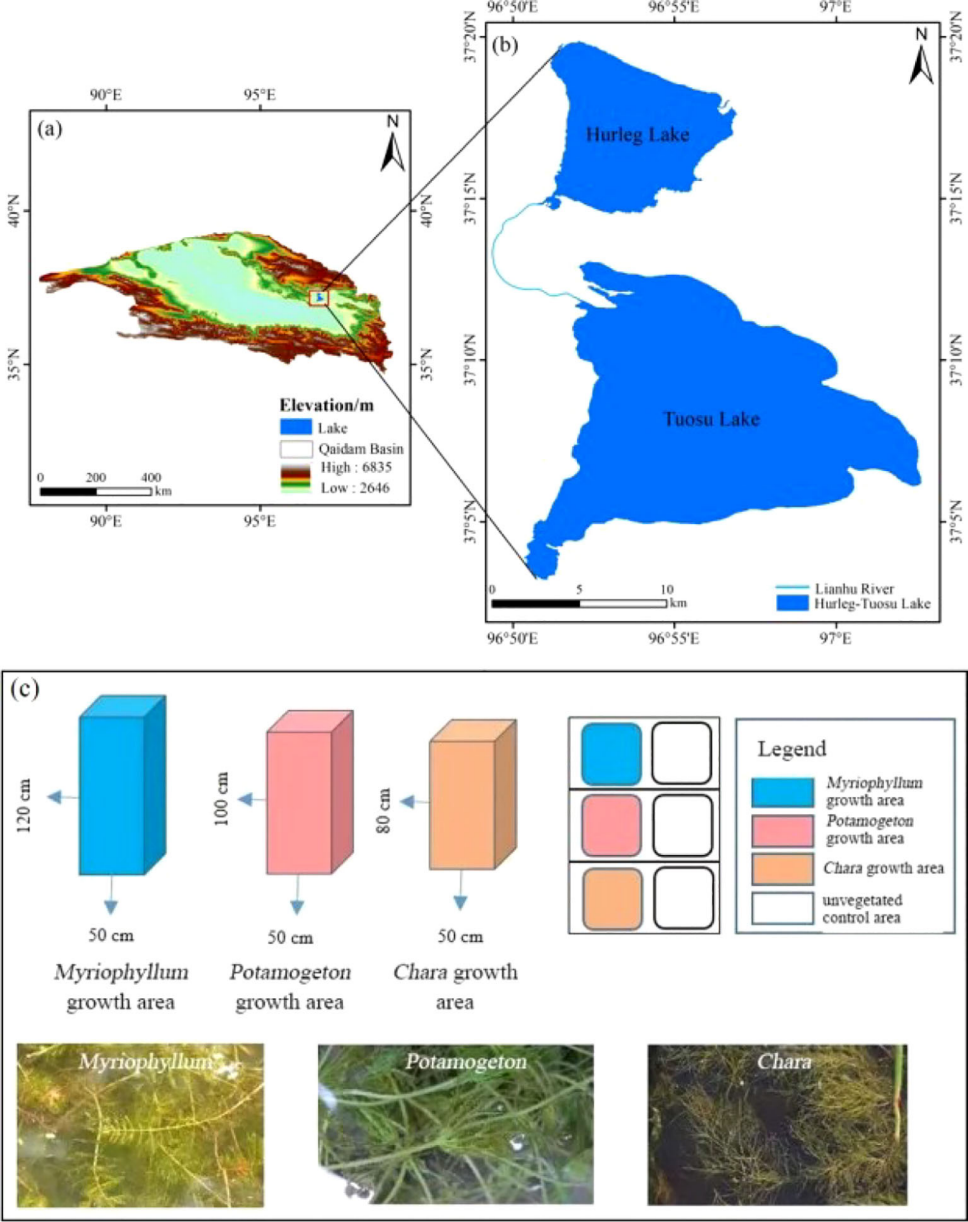
Geographical location and setting. **(A)** Location of the Hurleg Lake. **(B)** Hurleg Lake, its surrounding physical environments. **(C)** Schematic diagram of experimental design. Unvegetated control area, referring to an aquatic environment with consistent water depth but lack of the corresponding vegetation.

Hurleg Lake is located in the Qaidam Basin on the northeastern Qinghai-Tibetan Plateau, China (36°53′~38°11′N, 96°30′~97°50′E) (**Figure 2A**) (Shao et al., 2024). The region has an average annual temperature of 3.9°C, an average annual precipitation of 169.3 mm, and an average annual evaporation of 2036.3 mm, characterized by a typical cold and arid continental climate (Wang, 2017). Hurleg Lake has a surface area of 56.7 km^2^ and an average depth of 2.9 m, making it the largest freshwater lake in the Qaidam Basin (Wen et al., 2021; Wang et al., 2016). The lake is primarily recharged by the Bayin River and the Balegen River from upstream and flows into the downstream Tuosu Lake via the Lianshui River (**Figure 2B**) (Sun et al., 2023). Field investigations revealed that Hurleg Lake contains abundant aquatic plants such as *Myriophyllum*, *Chara*, and *Potamogeton*, with extensive reed beds surrounding the lake, classifying it as a typical shallow macrophyte-dominated lake.

### Sample collection and data analysis

#### Experimental design

Based on preliminary investigations, experimental devices were deployed to simulate the effects of the growth of *Chara*, *Myriophyllum*, and *Potamogeton* on the carbon exchange processes within the Hurleg lake. The experimental devices are placed near the lake, 3 m away from the lake. Specifically, transparent acrylic columns with a bottom diameter of 50 cm and open tops were set up along the lakeshore. A 20 cm layer of sediment-collected from the corresponding plant growth area-was evenly placed at the bottom of each column. Intact and undamaged specimens of *Chara*, *Myriophyllum*, and *Potamogeton*, along with lake water from their respective growth areas, were introduced into the columns.

According to the actual water depths at which these plants grow in the lake, the water levels in the cylinders containing *Myriophyllum*, *Potamogeton*, and *Chara* were maintained at 120 cm, 100 cm, and 80 cm, respectively (**Figure 2C**). Additionally, one unvegetated acrylic tank was set up as the unvegetated control group. To prevent light penetration through the cylinder walls from affecting the sediment, the outer surface of each experimental setup was wrapped with aluminum foil to simulate the low-light conditions of natural surface sediments in the field. Throughout the experiment, *in-situ* lake water was added as needed to maintain the overlying water at the target depth. All devices were placed outdoors under natural light conditions. The aquatic plants were pre-cultured for two days to stabilize growth and allow the water in the cylinders to clear before sampling commenced.

### Sample collection and analysis

#### Water-air interface greenhouse gas sampling

The exchange fluxes of greenhouse gases at the water-air interface were collected and measured using the static chamber-gas chromatography method. The static chamber was constructed from polycarbonate (PC) material into a cylindrical shape with a diameter of 26 cm and a height of 35 cm, featuring a sealed top and an open bottom. A moisture-proof mat was attached to the bottom to allow the chamber to float on the water surface. The top of the chamber was fitted with three short tubes: one connected to a gas sampling line ending with a syringe equipped with a three-way valve; one holding an electronic thermometer; and one containing an electric fan inside the chamber to ensure homogeneous mixing of the air (Lin et al., 2023; Liu et al., 2024).

Gas sampling was conducted at 0, 4, 8, 12, 16, 20, and 24 hours after the initiation of enclosure on July 19, August 10, August 21, and October 4, 2024. Each time, a 50 mL gas sample was collected using a syringe with a three-way valve. After collection, all gas samples were transported to the laboratory for concentration analysis using a gas chromatograph (Agilent 7890B, USA). The greenhouse gas fluxes were finally calculated using formula 1:

(1) $$F=\text{ρ}\times \frac{\text{V}}{\text{A}}\times \frac{\text{P}}{{\text{P}}_{0}}\times \frac{{\text{T}}_{0}}{\text{T}}\times \frac{{\text{dC}}_{\text{t}}}{\text{dt}}$$

Where *F* is the greenhouse gas emission flux (mg·m^-2^·h^-1^); ρ is the density of the target gas under standard conditions (g/L); V is the volume of the static chamber (m^3^); A is the area covered by the static chamber (m^3^); P is the atmospheric pressure at the sampling site (hPa); P_0_ is the standard atmospheric pressure (hPa); T_0_ is the absolute temperature under standard conditions (K); T is the absolute temperature inside the chamber during sampling (K); dC_t_/d_t_ is the rate of change in gas concentration over time within the chamber.

A positive value of *F* indicates that the water body is emitting greenhouse gases into the atmosphere, whereas a negative value signifies absorption from the atmosphere.

### Water-sediment interface greenhouse gas sampling

The exchange flux of greenhouse gases at the water-sediment interface was simulated using an *in-situ* sediment simulation method (Cowan and Boynton, 1996), conducted within the same acrylic cylinders as those used for the water-air interface greenhouse gas flux experiments. The procedure consisted of the following steps: a syringe connected to an extension tube was used to extract overlying water, with the end of the tube placed 3–5 cm above the sediment surface; each extraction collected 30 mL of water. Simultaneously, Rhizon pore-water samplers were employed to collect sediment interstitial water to investigate its influence on greenhouse gas exchange fluxes at the water-sediment interface. The Rhizon sampler consists of a porous hydrophilic polyethersulfone (PES) tip (2.5 mm in diameter, 50 mm in length, 0.6 µm pore size). The sampler connected to a vacuum syringe was carefully inserted into the sediment to collect pore water (30–40 mL); all procedures were performed under airtight conditions to prevent gas exchange.

Both the overlying water and interstitial water samples were immediately subjected to headspace equilibration treatment on-site. Specifically, an equal volume of high-purity nitrogen gas was injected into the water samples at a 1:1 ratio, followed by vigorous shaking for 3 minutes to achieve thorough gas-water mixing. The equilibrated gas in the headspace was then extracted using a gas-tight syringe, sealed, labeled, and transported to the laboratory for determination of greenhouse gas concentrations via gas chromatography (Agilent 7890B, USA).

Sampling of greenhouse gases at the water-sediment interface was conducted synchronously with the sampling at the water-air interface. Based on the analytical results from the gas chromatograph, the greenhouse gas exchange flux at the water-sediment interface was calculated using Formula (Equation 2):

(2) F=ΔC×V/(A×Δt)

Where *F* represents the greenhouse gas exchange flux at the water-sediment interface (mg·m^-2^·h^-1^); ΔC denotes the change in greenhouse gas concentration before and after incubation (mg/L); V is the volume of overlying water in the incubation tube (L); A is the cross-sectional area of the incubation tube (m^2^); Δt is the incubation time (h).

A positive value of *F* indicates that greenhouse gases are released from the sediment into the overlying water, while a negative value signifies absorption of greenhouse gases by the sediment.

To evaluate the reliability of the CO_2_ flux results, duplicate samples were collected simultaneously with the actual samples at 12:00 on July 19, 16:00 on August 21, and 20:00 on October 4. The calculated absolute differences in *F*(CO_2_) between the duplicate and actual samples ranged from 0.02 to 3.51 mg·m^–2^·h^–1^ for the water-air interface and from 0.07 to 8.73 mg·m^–2^·h^–1^ for the water-sediment interface. Further analysis revealed that the relative deviations of the parallel samples were 0.45%-17.36% for the water-air interface and 0.66%-11.22% for the water-sediment interface. All relative deviations of the parallel samples were below the standard threshold of 20%, indicating stable measurement procedures and good data reliability in this study.

Simultaneous to sample collection, meteorological and aquatic environmental parameters were recorded as supporting background data. Meteorological variables-including wind speed, air temperature, and atmospheric pressure-were measured using a handheld weather meter (Kestrel 5500). Key aquatic environmental factors, such as water temperature, pH, DO, electrical conductivity (EC), total dissolved solids (TDS), and oxidation-reduction potential (ORP), were determined using a multiparameter water quality probe (Aquaread AP-1000).

### Data analysis and visualization

Data processing, statistical analysis, and graph generation were performed using Excel 2013 and Origin 2024. The geographic overview map of the study area was created with ArcGIS 10.8. To quantify the explanatory contributions of environmental variables to *F*(CO_2_) at both the water-air and water-sediment interfaces, redundancy analysis (RDA) was employed. This method helped identify the primary influencing factors. The RDA ordination plot was generated using the software Canoco 5.

## Results

### Diurnal variations in meteorological conditions and water environmental parameters in Hurleg Lake

**Figure 3:**
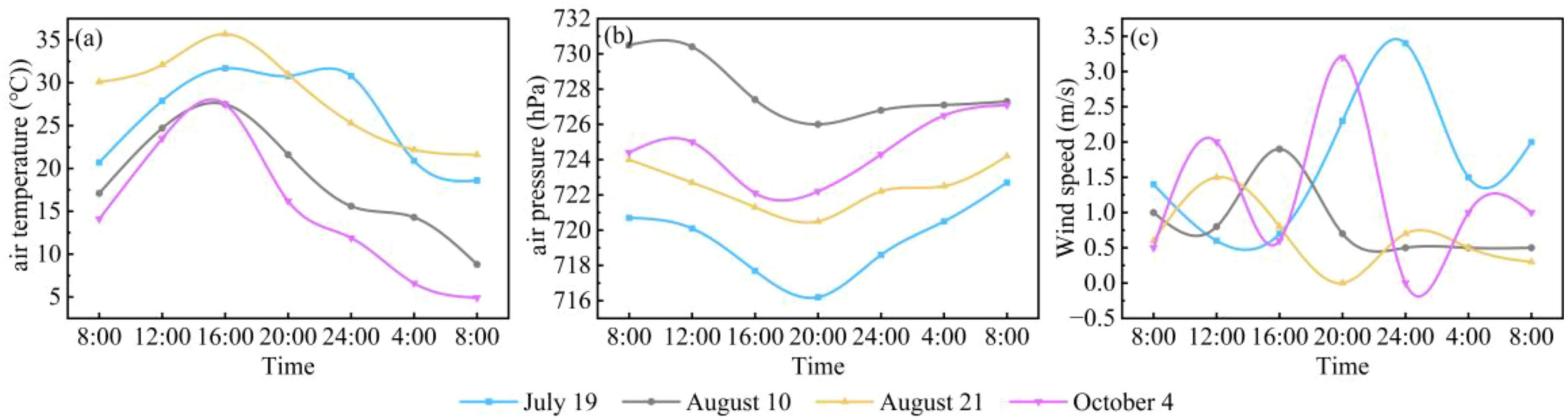
Diurnal variations in meteorological conditions in Hurleg Lake. **(A)** air temperature; **(B)** air pressure; **(C)** wind speed.

During the monitoring period, air temperature, atmospheric pressure, and wind speed at Hurleg Lake all exhibited significant diurnal variations (**Figure 3**). Air temperature showed a consistent pattern of increasing after sunrise, reaching its peak around 16:00, then gradually decreasing and rising again after 04:00 the next day (**Figure 3A**). Atmospheric pressure exhibited a trend of decreasing first and then increasing, with the maximum values generally occurring around 08:00 and the minimum values appearing around 20:00 (**Figure 3B**). Wind speed fluctuated considerably, with values ranging from 0 to 3.4 m/s and an average of 1.09 m/s (**Figure 3C**).

**Figure 4:**
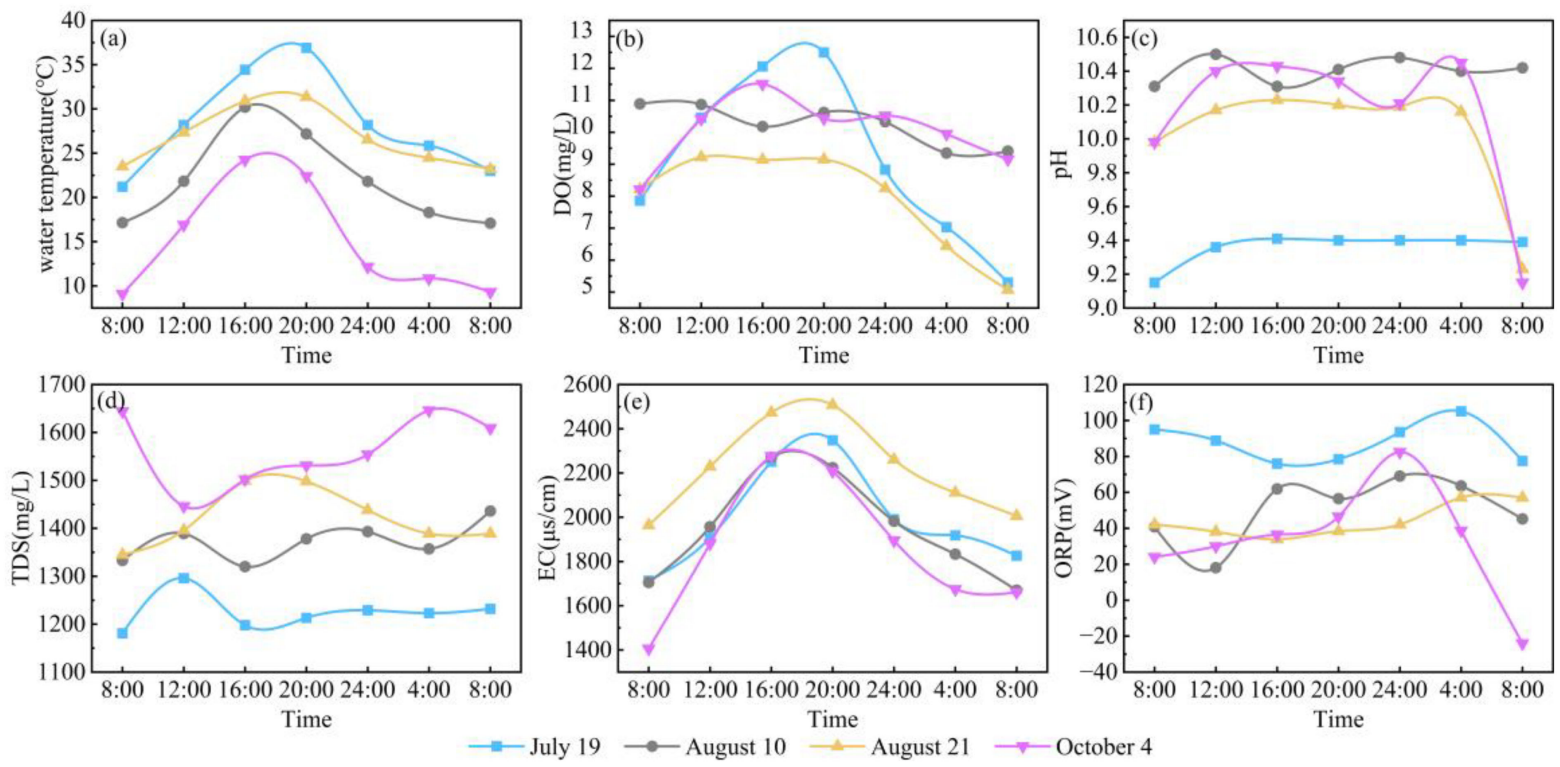
Diurnal variations in water environmental parameters in Hurleg Lake. **(A)** water temperature; **(B)** DO; **(C)** pH; **(D)** TDS; **(E)** EC; **(F)** ORP.

During the monitoring period, the water temperature at Hurleg Lake exhibited a pattern of initial increase followed by a decrease throughout the day. The lowest water temperature occurred before sunrise, while the highest was observed around 16:00, with values ranging from 9.1 to 36.9 °C (**Figure 4A**). DO showed a trend of first increasing and then decreasing, reaching its maximum around 20:00 and its minimum around 08:00 the next day. Overall, DO varied between 5.07 and 12.5 mg/L, with an average value of 9.33 mg/L (**Figure 4B**). The pH fluctuated within a range of 9.15 to 10.48, averaging 9.98 (**Figure 4C**). TDS ranged from 1181 to 1646 mg/L, with a mean value of 1395 mg/L (**Figure 4D**). EC increased sharply starting at 08:00, peaked between 16:00 and 20:00, and then decreased significantly. Overall, EC values varied from 1406 to 2507 μs/cm, with an average of 2005 μs/cm (**Figure 4E**). ORP showed considerable diurnal variation, with the lowest values occurring around 08:00 and the highest around 00:00. ORP ranged from -24 to 105.1 mV, with an average of 54.01 mV (**Figure 4F**).

### Diurnal variation in *F*(CO2) at the water-air and water-sediment interfaces in Hurleg Lake

**Figure 5:**
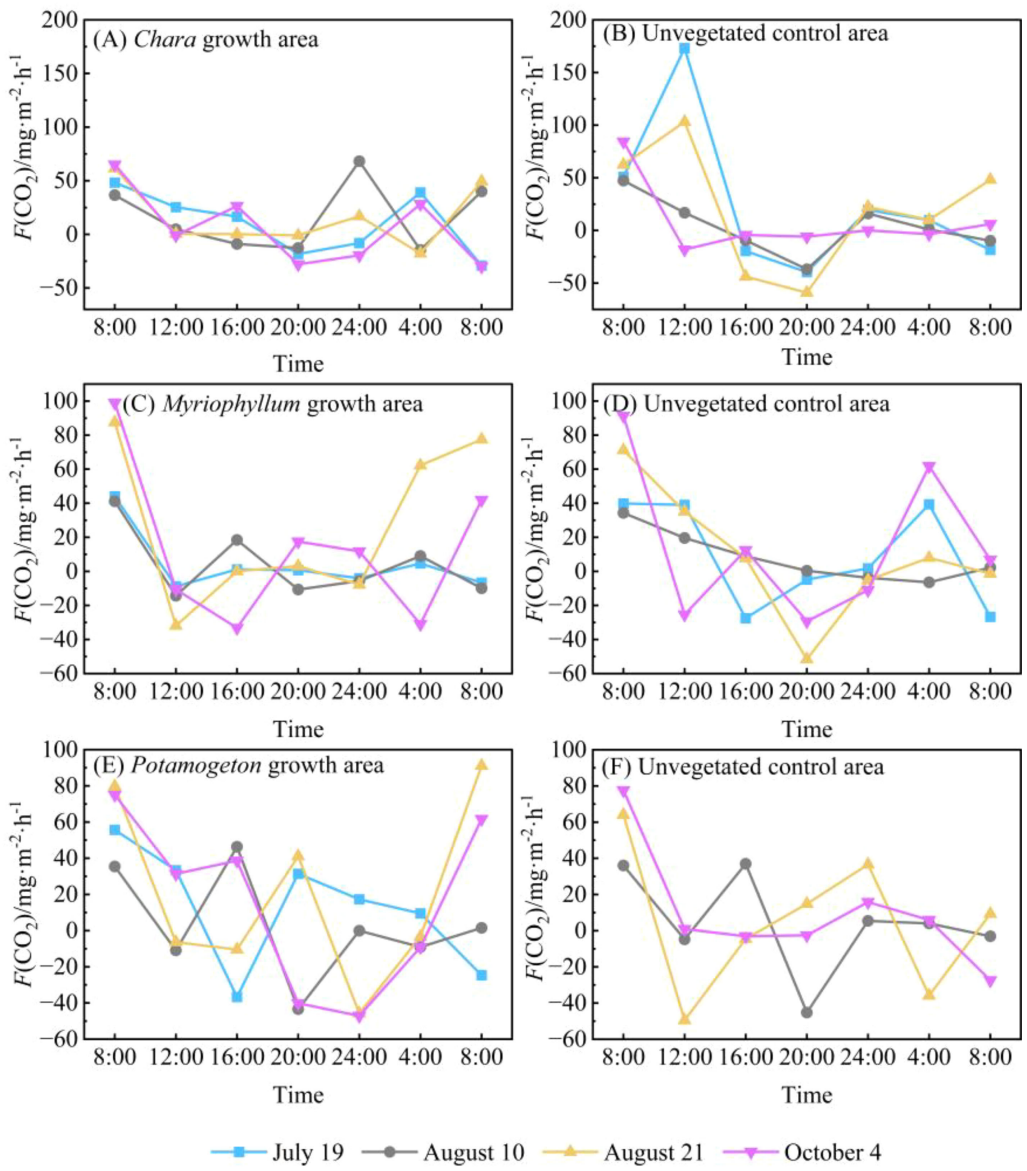
Diurnal variation in *F*(CO_2_) at the water-air interfaces in Hurleg Lake. **(A)***F*(CO_2_) in the *Chara* growth area; **(B)***F*(CO_2_) in the unvegetated *Chara* area; **(C)***F*(CO_2_) in the *Myriophyllum growth area*; **(D)***F*(CO_2_) in the unvegetated *Myriophyllum* area; **(E)***F*(CO_2_) in the *Potamogeton* growth area; **(F)***F*(CO_2_) in the unvegetated *Potamogeton* area.

During the four monitoring days, the *F*(CO_2_) at water-air interface in the *Chara* growth area of Hurleg Lake exhibited similar diurnal variation patterns. Peak emission fluxes consistently occurred around 08:00, while values remained relatively low between 12:00 and 20:00 (**Figure 5A**). In the unvegetated control area, the maximum value of *F*(CO_2_) at the water-air interface generally appeared around 12:00 (**Figure 5B**). The highest flux during the monitoring period was 173.18 mg·m^-2^·h^-1^, recorded at 12:00 on July 19. The minimum flux values in both the *Chara* growth area and the unvegetated control area occurred at approximately the same time.

The *F*(CO_2_) at water-air interface in the *Myriophyllum* growth area showed relatively consistent variation trends during the monitoring period, with peaks occurring at 08:00, followed by consistently low levels from 12:00 to 24:00 (**Figure 5C**). The overall variation range of *F*(CO_2_) in this area was -33.22 to 99.02 mg·m^-2^·h^-1^, with a mean value of 12.33 mg·m^-2^·h^-1^. The variation pattern of *F*(CO_2_) in the unvegetated control area was relatively complex: while the peak occurred at the same time as in the *Myriophyllum* growth area and dropped to a trough around 20:00, the overall variation ranged from -51.58 to 91.34 mg·m^-2^·h^-1^, with an average of 10.25 mg·m^-2^·h^-1^(**Figure 5D**). The variation trends of *F*(CO_2_) in both the *Potamogeton* growth area and its unvegetated control area were the most complex among all monitored areas, showing large fluctuations (**Figures 5E, F**). Peaks in both areas consistently occurred around 08:00; however, the timing of the troughs showed no consistent pattern.

**Figure 6:**
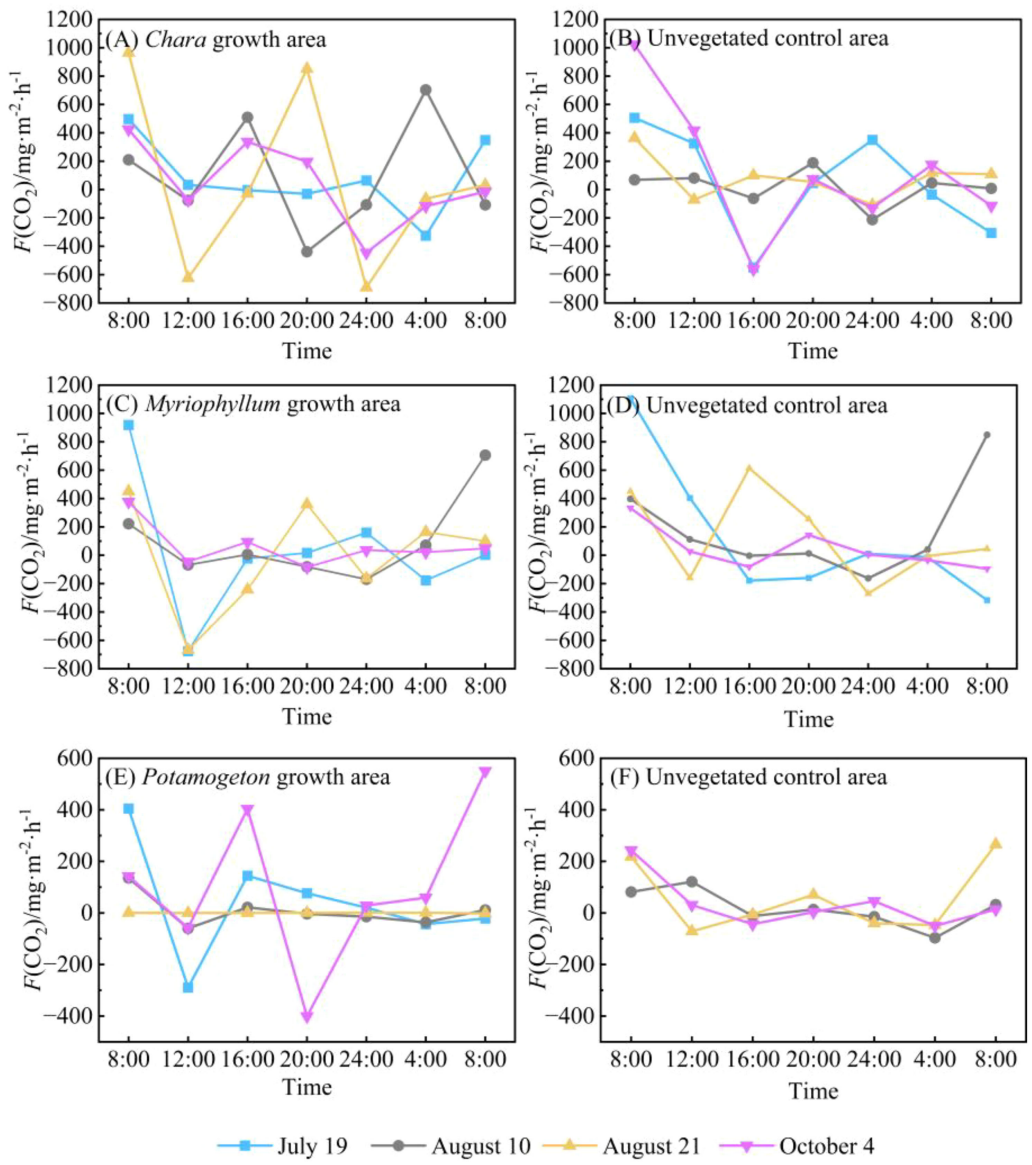
Diurnal variation in *F*(CO_2_) at the water-sediment interfaces in Hurleg Lake. **(A)***F*(CO_2_) in the *Chara* growth area; **(B)***F*(CO_2_) in the unvegetated *Chara* area; **(C)***F*(CO_2_) in the *Myriophyllum growth area*; **(D)***F*(CO_2_) in the unvegetated *Myriophyllum* area; **(E)***F*(CO_2_) in the *Potamogeton* growth area; **(F)***F*(CO_2_) in the unvegetated *Potamogeton* area..

In contrast to the water-air interface, the *F*(CO_2_) at the water-sediment interface exhibited a wider range of fluctuations (**Figure 6**). The water-sediment interface *F*(CO_2_) in the *Chara* growth area showed strong diurnal variability with considerable amplitude, emitting peak values around 08:00 and reaching troughs around 24:00 (**Figure 6A**). Throughout the monitoring period, *F*(CO_2_) varied widely from -690.77 to 964.86 mg·m^-2^·h^-1^, with a mean value of 72.24 mg·m^-2^·h^-1^. The variation pattern of *F*(CO_2_) at the water-sediment interface in the corresponding unvegetated control area was similar to that in the *Chara* growth area, with emission peaks also occurring around 08:00 (**Figure 6B**). However, two distinct troughs were observed at 16:00 and 24:00, and the overall amplitude of variation was relatively smaller.

Similarly, the *F*(CO_2_) at the water-sediment interface in the *Myriophyllum* growth area peaked around 08:00, with a distinct trough observed around 12:00. During the remaining periods, the fluxes remained relatively stable (**Figure 6C**). The *F*(CO_2_) in this area ranged from -676.83 to 918.76 mg·m^-2^·h^-1^, with a mean value of 48.39 mg·m^-2^·h^-1^. In contrast, the unvegetated control area adjacent to the *Myriophyllum* growth area showed smaller fluctuations in *F*(CO_2_) at the water-sediment interface, though its mean value (118.72 mg·m^-2^·h^-1^) was higher than that of the vegetation area. Peak CO_2_ emissions in this control area occurred around 08:00, with the lowest values appearing around 24:00 (**Figure 6D**). The variation patterns of *F*(CO_2_) at the water-sediment interface in the *Potamogeton* growth area differed noticeably across monitoring dates (**Figure 6E**). The fluxes varied from -289.15 to 404.86 mg·m^-2^·h^-1^, averaging 23.62 mg·m^-2^·h^-1^. Emission peaks generally occurred around 08:00, with additional peaks observed around 12:00 and 20:00 on some monitoring days. In the corresponding unvegetated control area, CO_2_ emission peaks mainly appeared around 08:00, and consistently exhibited two distinct troughs around 16:00 and 04:00 throughout the monitoring period.

During the four monitoring days, the *F*(CO_2_) at both the water-air and water-sediment interfaces showed positive values (indicating net CO_2_ emissions) during daytime across all dates and in all vegetated and unvegetated control areas, with one exception: the water-sediment interface in the *Myriophyllum* growth area recorded a negative value (acting as a CO_2_ sink) on August 21 (**Table 1**). These results demonstrate that, throughout the monitoring period, both interfaces generally functioned as net sources of CO_2_ (carbon sources) during daytime.

**Table 1:** Diurnal and daily mean values of *F*(CO_2_) at the water-air and water-sediment interfaces of Hurleg Lake.

Date	Plant area	*F*(CO_2_) at water-air (mg·m^-2^·h^-1^)			*F*(CO_2_) at water-sediment (mg·m^-2^·h^-1^)		
		Daytime	Nighttime	Daily average	Daytime	Nighttime	Daily average
July 19	*Chara* growth area	17.98	-4.1	10.54	124.16	14.45	83.54
	Unvegetated control area	41.27	-0.72	25.14	82.16	13.99	48.36
	*Myriophyllum* growth area	9.27	-1.33	4.43	58.87	0.7	31.7
	Unvegetated control area	11.63	2.4	8.71	293.17	-119.69	121.97
	*Potamogeton* growth area	20.94	8.4	12.28	84.14	7.99	41.71
	Unvegetated control area						
August 10	*Chara* growth area	4.95	20.26	16.22	51.02	12.45	98.93
	Unvegetated control area	4.46	-7.43	3.58	68.91	7.85	16.94
	*Myriophyllum* growth area	8.67	-4.29	4.02	19.35	131.32	97.74
	Unvegetated control area	15.80	-1.89	7.90	129.87	184.93	178.05
	*Potamogeton* growth area	6.87	-12.75	2.84	23.38	-10.82	7.69
	Unvegetated control area	5.75	-9.74	4.19	50.89	-16.35	17.84
August 21	*Chara* growth area	15.33	11.89	15.69	290.52	31.91	62.62
	Unvegetated control area	15.71	5.36	20.48	111.67	42.95	80.62
	*Myriophyllum* growth area	14.70	33.75	27.25	-24.88	115.06	0.20
	Unvegetated control area	15.61	-12.52	9.13	289.79	6.24	132.64
	*Potamogeton* growth area	25.99	20.79	20.87	0.22	0.12	0.13
	Unvegetated control area	6.21	6.21	4.98	52.84	62.43	55.81
October 4	*Chara* growth area	15.66	-12.30	5.91	221.03	-95.05	43.86
	Unvegetated control area	14.05	-0.81	8.41	237.90	1.22	125.98
	*Myriophyllum* growth area	18.17	10.05	13.63	85.41	5.49	63.92
	Unvegetated control area	12.29	7.07	15.25	105.37	4.02	42.22
	*Potamogeton* Growth Area	26.23	-8.67	15.77	21.83	59.56	103.75
	Unvegetated control area	18.23	-2.05	9.61	58.49	3.24	34.81

## Discussion

### Diel dynamic characteristics of *F*(CO_2_) in shallow macrophyte-dominated lakes

This study found that in Hurleg Lake, the *F*(CO_2_) at both the water-air and water-sediment interfaces exhibited significant diel dynamic characteristics. Peaks were generally concentrated around 08:00 in the early morning, while troughs mostly occurred around 20:00 in the evening and 24:00 at night. This diel dynamic pattern is consistent with findings from studies on other shallow macrophyte-dominated lakes in China (Sun et al., 2022; Zhang and Gao, 2021).

The diurnal differences in *F*(CO_2_) in shallow macrophyte-dominated lakes mainly originate from the daily alternation of photosynthesis and respiration. In the vegetated areas of Hurleg Lake, although submerged macrophytes absorb CO_2_ through photosynthesis during the daytime, the amount absorbed remains lower than the total CO_2_ released by nocturnal respiration and the degradation of organic matter in the water body. As a result, during daytime *F*(CO_2_) overall still exhibits net release. At night, plants may actively absorb CO_2_ through Crassulacean Acid Metabolism (CAM) or aerenchyma (Zhang, 2021; Keeley, 1998), while part of the CO_2_ produced by respiration is offset by organic carbon storage mechanisms, leading to brief periods of CO_2_ uptake. In the unvegetated control areas, daytime is primarily dominated by continuous microbial respiration releasing CO_2_; at night, decreased water temperature inhibits microbial activity, reducing CO_2_ production. Together with enhanced water stability and weakened vertical mixing, resulted in lower surface *ρ*CO_2_, and thereby causing CO_2_ absorption.

### Regulation of sediment CO_2_ release by submerged macrophyte type

Organic matter in lake sediments is converted into CO_2_ through decomposition and mineralization, released into the overlying water, and then diffuses into the atmosphere through the water-air interface (Tang and Zhang, 2023). This study calculated the ratio of *F*(CO_2_) at the water-air interface to that at the water-sediment interface (Ratio a) to characterize the relative contribution of sediment-released CO_2_ to the net atmospheric flux (**Table 2**). The results indicate that different types of submerged macrophytes significantly regulate the release of CO_2_ from sediments. Among the vegetated areas, the *Potamogeton* growth area had the highest Ratio a (25.24%), indicating a weaker capacity for CO_2_ retention, allowing more CO_2_ to escape to the atmosphere; the *Chara* growth area had the lowest (14.34%), showing a stronger capacity for CO_2_ retention or transformation. Among the unvegetated control areas, the *Myriophyllum* control area, despite having an extremely high sediment CO_2_ flux (118.72 mg·m^-2^·h^-1^), had a Ratio a of only 7.95%, suggesting the presence of a strong CO_2_ sink mechanism in this area where the vast majority of sediment-released CO_2_ is retained within the water body, potentially used for aquatic organism metabolism or transformed into other forms of carbon.

**Table 2:** Mean *F*(CO_2_) and relative contributions at the water-air and sediment-water interfaces across vegetated areas and unvegetated controls areas.

Plant area	Mean *F*(CO_2_) at the water-air interface(mg·m^-2^·h^-1^)	Mean *F*(CO_2_) at the water-sediment interface (mg·m^-2^·h^-1^)	Ratio a (%)	Ratio b (%)	Ratio c (%)
*Chara* growth area	12.09	72.24	14.34%	22.61%	5.57%
Unvegetated control area	14.40	67.98	17.48%	5.00%	11.65%
*Myriophyllum* growth area	12.33	48.39	20.31%	42.90%	64.54%
Unvegetated control area	10.25	118.72	7.95%	9.79%	8.45%
*Potamogeton* growth area	12.94	38.32	25.24%	8.85%	30.50%
Unvegetated control area	4.69	27.12	14.75%	22.65%	23.31%

Ratio “a” = [Mean *F*(CO_2_) at the water-air interface]/[Mean *F*(CO_2_) at the water-air interface + Mean *F*(CO_2_) at the water-sediment interface] × 100%, reflecting the relative contribution of sediment-released CO^2^ to the net atmospheric emission flux of the water body; Ratio “b” represents the proportion of nocturnal *F*(CO_2_) to the diurnal total flux at the water-air interface in each vegetated and corresponding unvegetated control area; Ratio “c” indicates the proportion of nocturnal *F*(CO_2_) to the diurnal total flux at the water-sediment interface in each vegetated and corresponding unvegetated control area.

In Hurleg Lake, different types of submerged macrophytes significantly regulate the release of CO_2_ from sediments. This finding is consistent with studies on other shallow macrophyte-dominated lakes (**Table 3**). Cao’s (2022) research on Dongping Lake also showed significant differences in sediment CO_2_ production rates and interface fluxes among areas with different vegetation types (reed area, *Potamogeton* area, mixed growth area, and bare sediment area), further confirming that aquatic plant type is an important factor regulating the lake carbon cycle process. This also implies the potential for managing aquatic plant community structure to regulate the lake carbon cycle in the future. Furthermore, nocturnal CO_2_ exchange may play an important role in maintaining the carbon source function of shallow macrophyte-dominated lakes. Its proportion of the total diel flux reached 22.65% to 42.90% at the water-air interface and 5.57% to 64.54% at the water-sediment interface across various areas (Ratios b, c). Neglecting nocturnal fluxes would significantly underestimate the carbon emission intensity of shallow macrophyte-dominated lakes and increase the uncertainty in carbon budget assessment.

**Table 3:** Comparison of diurnal dynamics of CO_2_ exchange fluxes in different shallow macrophyte-dominated lakes.

Lake name	Lake type	Diel dynamic characteristics	Main driving factors	Lake name
Hurleg Lake	Shallow macrophyte lake	*F*(CO_2_) peaks around 08:00, troughs around 20:00 and 24:00	Water temperature, pH, DO, pressure, submerged plant type	This study
Capitol Lake	Shallow macrophyte lake	The highest *F*(CO_2_) occurs in early morning, gradually decreases, reaches trough after sunset	Solar radiation, lake trophic status, water temperature	Yang et al., 2019
Wuliangsu Lake	Shallow eutrophic macrophyte lake	The highest *F*(CO_2_) occurs before sunrise, and the lowest *F*(CO_2_) occurs in the afternoon	Algal blooms, submerged vegetation, weather, DO, temperature	Zhang, 2021
Taihu Lake	Shallow eutrophic macrophyte lake	Spring: the *F*(CO_2_) peaks around sunrise, troughs around 14:30; Summer: the *F*(CO_2_) peaks around sunrise and troughs afternoon	Air temperature, pressure, wind speed, solar radiation control	Jia et al., 2018

### Regulation of Hurleg Lake CO_2_ exchange flux by environmental factors

The magnitude of greenhouse gas exchange flux at the lake water-air interface is jointly regulated by production and transport processes (Yang and Tong, 2015) and is closely related to a series of physical and chemical processes. Specifically, CO_2_ in the water body primarily originates from the mineralization and decomposition of sedimentary organic matter and the respiration of aquatic plants (Gao and Yu, 2020), while the photosynthesis of aquatic plants and the precipitation of authigenic carbonates within the lake consume CO_2_ in the water (Jiang et al., 2012). These processes may collectively determine the dynamic balance of CO_2_ in the water body and its exchange flux at the water-air interface.

**Figure 7:**
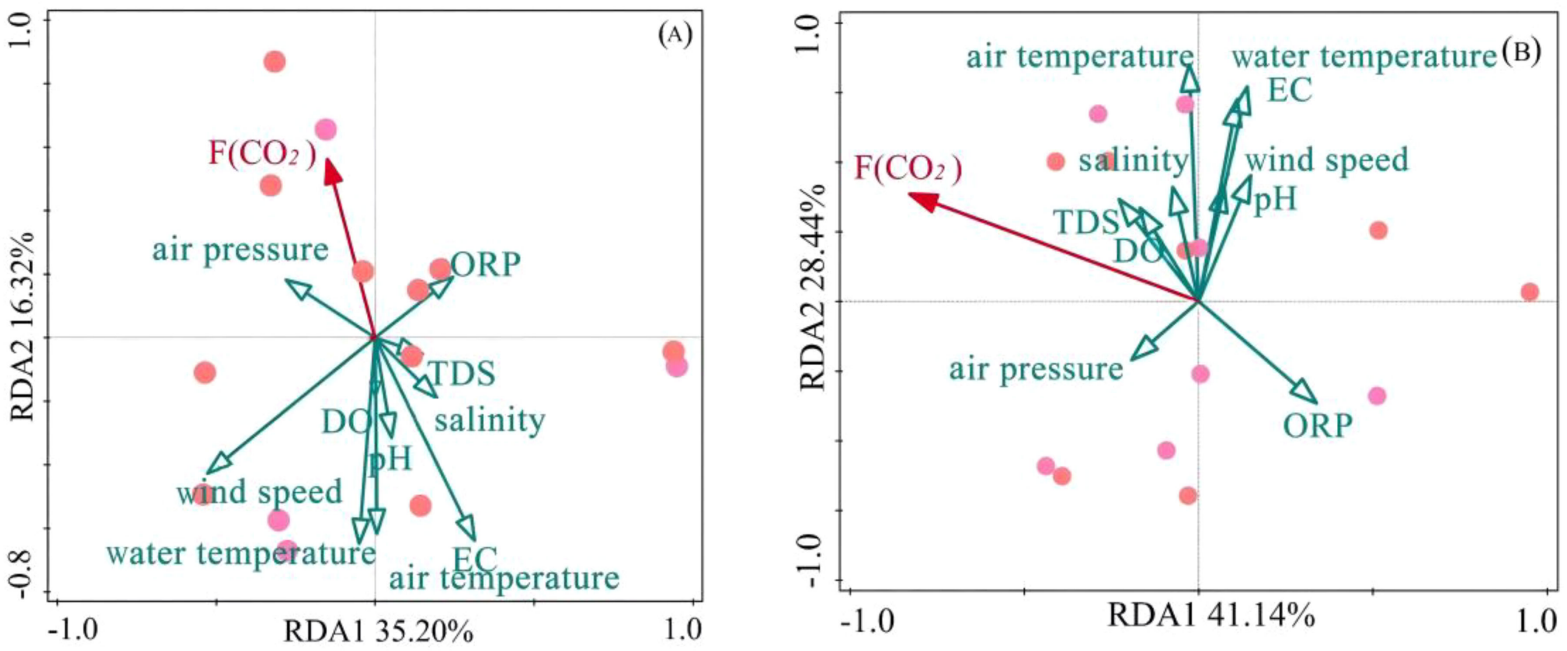
Redundancy analysis (RDA) biplots of CO₂ exchange fluxes and environmental factors in Hurleg Lake. **(A)** Water-air interface; **(B)** Water-sediment interface.

Water temperature is one of the key factors influencing *F*(CO_2_). Typically, higher water temperatures enhance photosynthesis in aquatic organisms, promoting the absorption of dissolved CO₂ in the water, thereby reducing ρCO_2_ in the water and increasing the water’s uptake of atmospheric CO_2_ (Tremblay et al., 2005). Therefore, the *F*(CO_2_) at water-air interface usually shows a negative correlation with water temperature (**Figure 7A**). Additionally, the pH value of the water body also significantly affects CO_2_ exchange flux. When water pH>8, free CO_2_ in the water converts to carbonate, leading to CO_2_ undersaturation in the water and consequently lower ρCO_2_. This state of low ρCO_2_ facilitates the entry of atmospheric CO_2_ into the water body (Wang et al., 2012), hence the *F*(CO_2_) at water-air interface also shows a negative correlation with pH. Changes in atmospheric pressure also significantly influence *F*(CO_2_) at water-air interface. When atmospheric pressure increases, the solubility of CO_2_ in water increases, which promotes the decomposition of organic carbon by aerobic bacteria, producing more CO_2_ (Yang et al., 2019), consequently leading to more CO_2_ release from the water body to the atmosphere. Therefore, *F*(CO_2_) at water-air interface shows a positive correlation with atmospheric pressure. Furthermore, enhanced photosynthesis by aquatic plants leads to increased DO concentration in the water, which in turn strengthens the water’s CO_2_ absorption capacity (Li et al., 2023), causing DO exhibits a negative correlation with *F*(CO_2_) at water-air interface.

Sediment is a source of CO_2_ for the water body. The CO_2_ concentration in sediment porewater directly influences the intensity of CO_2_ diffusion at the water-sediment interface (Sun et al., 2022). Higher concentrations of CO_2_ in sediment porewater accelerate CO_2_ production at the sediment surface and promote the release of CO_2_ from the sediment into the overlying water (Ometto et al., 2013). Besides porewater CO_2_ concentration, the size and connectivity of sediment pores are also key parameters controlling the migration of CO_2_ from sediment to overlying water (Gudasz et al., 2010). Larger pores reduce the resistance to upward diffusion of CO_2_, improving diffusion efficiency, while highly connected pores form continuous pathways for CO_2_ diffusion, making it easier for CO_2_ to pass through the sediment into the water body. Additionally, higher temperatures promote the rate of organic carbon mineralization, thereby producing more CO_2_ (Yang et al., 2019), leading to increased CO_2_ concentration and facilitating the diffusion of CO_2_ from the sediment into the overlying water (Grasset et al., 2018). Therefore, *F*(CO_2_) shows a positive correlation with water temperature and air temperature (**Figure 7B**). During the monitoring of *F*(CO_2_) at the water-air and water-sediment interfaces in Hurleg Lake, both positive and negative correlations were observed between *F*(CO_2_) and factors such as water temperature, pH, and atmospheric pressure. However, existing studies confirm that in complex interface systems, *F*(CO_2_) is often regulated by the nonlinear superposition of multiple factors rather than a single linear relationship (Cole et al., 1994).

### Carbon source/sink function of shallow macrophyte-dominated lakes and their environmental response

Hurleg Lake overall behaved as a source of atmospheric CO_2_ during the monitoring period, a finding consistent with many studies on shallow macrophyte-dominated lakes. However, the carbon source/sink function of shallow macrophyte-dominated lakes is not fixed but exhibits significant temporal dynamics and spatial heterogeneity. Climate change and human activities are impacting the carbon cycle in shallow macrophyte-dominated lakes. Climate warming may promote a shift of shallow macrophyte-dominated lakes towards being carbon sinks, while eutrophication may enhance this trend (Hu, 2020). However, this response may vary depending on lake type and environmental conditions, requiring more research to deeply understand the response and adaptation mechanisms of the carbon cycle in shallow macrophyte-dominated lakes to global change.

## Conclusions

This study selected Hurleg Lake-a typical shallow macrophyte-dominated lake in the northeastern Qinghai-Tibetan Plateau-as a case study to investigate diurnal variations in *F*(CO_2_) at water-air and water-sediment interface and their influencing factors. The *F*(CO_2_) exhibit significant diel dynamics characterized by early morning peaks and troughs in the evening and late night, a pattern may regulated by the diurnal alternation of photosynthesis and respiration and representing a common feature of such lakes. The types of submerged macrophytes significantly influenced the carbon cycle, with varying contributions of sediment-released CO_2_ to atmospheric diffusion, probably owing to interspecific differences in photosynthetic efficiency, carbon concentration mechanisms, and root exudate composition. Nocturnal CO_2_ exchange was critical to the lake’s carbon source function, indicating that neglecting nighttime fluxes would greatly underestimate carbon emissions and increase uncertainty in carbon budget assessments. Furthermore, the *F*(CO_2_) at water-air interface correlated negatively with water temperature, pH, and dissolved oxygen but positively with atmospheric pressure, whereas water-sediment fluxes were primarily driven by sediment porewater CO_2_ concentration, sediment porosity, and water temperature, reflecting the complex multi-factor interactions governing CO_2_ dynamics in these ecosystems.

## Data Availability

The original contributions presented in the study are included in the article/supplementary material. Further inquiries can be directed to the corresponding author.
